# New drugs and stock market: a machine learning framework for predicting pharma market reaction to clinical trial announcements

**DOI:** 10.1038/s41598-023-39301-4

**Published:** 2023-08-07

**Authors:** Semen Budennyy, Alexey Kazakov, Elizaveta Kovtun, Leonid Zhukov

**Affiliations:** 1 Sber AI Lab, Moscow, Russia; 2 Artificial Intelligence Research Institute (AIRI), Moscow, Russia; 3 Higher School of Economics University, Moscow, Russia

**Keywords:** Computer science, Market analysis

## Abstract

Pharmaceutical companies operate in a strictly regulated and highly risky environment in which a single slip can lead to serious financial implications. Accordingly, the announcements of clinical trial results tend to determine the future course of events, hence being closely monitored by the public. Most works focus on retrospective analysis of announcement impact on company stock prices, bypassing the consideration of the problem in the predictive paradigm. In this work, we aim to close this gap by proposing a framework that allows predicting the numerical values of announcement-induced changes in stock prices. In fact, it is a problem of the impact prediction of the specific event on the corresponding time series. Our framework includes a BERT model for extracting the sentiment polarity of announcements, a Temporal Fusion Transformer for forecasting the expected return, a graph convolution network for capturing event relationships, and gradient boosting for predicting the price change. We operate with one of the biggest FDA (the Food and Drug Administration) datasets, consisting of 5436 clinical trial announcements from 681 companies for the years 2018–2022. During the study, we get several significant outcomes and domain-specific insights. Firstly, we obtain statistical evidence for the clinical result promulgation influence on the public pharma market value. Secondly, we witness inherently different patterns of responses to positive and negative announcements, reflected in a stronger and more pronounced reaction to negative clinical news. Thirdly, we discover two factors that play a crucial role in a predictive framework: (1) the drug portfolio size of the company, indicating the greater susceptibility to an announcement in the case of low diversification among drug products and (2) the announcement network effect, manifesting through an increase in predictive power when exploiting interdependencies of events belonging to the same company or nosology. Finally, we prove the viability of the forecast setting by getting ROC AUC scores predominantly greater than 0.7 for the classification of price change on historical data. We emphasize the transferability and generalizability of the developed framework on other datasets and domains but on the condition of the presence of two key entities: events and the associated time series.

## Introduction

The relation between the company market value and company events attracted the research community’s attention from the very moment it became technically possible to collect and merge the corresponding data. One of the pioneer works is considered to belong to Dolley, James Clay in 1933^1^. In practice, the study of events finds application in a wide range of socio-economic areas. In particular, the event studies are widely used within the interface between the law and economics to evaluate the impact of change in the regulatory environment on the company’s market value. In legal liability, it is exploited to assess damages^2^. In investment companies, event studies are widely used to form an investment strategy. In recent years, the pharmaceutical sector has become one of the most discussed because of COVID-19.

A biopharmaceutical company’s market value depends on two factors: a current product portfolio of the company with the intellectual rights associated with it and a portfolio of potential new drugs. Potential new drugs and clinical trials related to them directly influence the further market value behavior. The biopharmaceutical industry keeps high investment risks in research and development (R&D) due to high operation and capital costs, relatively high value of drug’s time-to-market (up to 15 years^3,4^), full dependence on regulatory agencies, high uncertainty^5^ in clinical trials, and ethical aspects^6^. Consequently, the market is highly sensitive to clinical trial results^7,8^. Besides, the late-phase clinical studies are the most complicated and expensive (Phase III takes up to 40% of total R&D costs^9^). Thus, understanding market value behavior for an upcoming clinical trial announcement is crucial to hedge potential financial risks, such as instant depreciation of market value, loss of stakeholders’ confidence, or even default.

The results of clinical trials of new medicines are supplied through official (company announcements, announcements of regulatory agencies) and non-official (mass media, pharma analytic agencies) sources. The most representative and widely exploited official sources of clinical announcements are the Food and Drug Administration (FDA), the European Medicines Agency (EMA), and other regulatory agencies providing the status of drug research and validation for certain areas (The Medicines and Healthcare products Regulatory Agency (MHRA), The Ministry of Health, Labour and Welfare, etc.).

The previous research^10^ covered such sources as EMA and specialized medical agencies. Perez-Rodriguez^11^ analyzed outliers in market indicators of several pharmaceutical companies and assigned the causes to such outliers based on research and development activities covering clinical trials, drug design, and scientific publications. Tomovic^12^ compared the impact of the FDA and dividend payment announcements on the market prices. In turn, the study^13^ identified characteristics analysis, which was indispensable for explaining the bio-pharmaceutical market reaction to announcements on product innovation. A couple of recent papers were focused on market influence during the pandemic. Rouatbi^14^ discussed the effect that vaccines have on market volatility and compared them for different regions of the world. With growing interest in advanced statistical approaches (e.g., machine learning, deep learning, time series analysis), event study is becoming less retrospective, and more attempts are aimed at building forecast models that evaluate the future behavior of market value for upcoming events^15,16^. The forecast models may support investors’ decisions and evaluate associate risk according to the previous statistics. Moreover, machine learning-powered systems can be used not only for financial goals. The authors^17,18^ investigated target diseases of FDA-approved drugs using network theory. Ridder^19^ anticipated outcomes of Phase III in the drug development process based on data from Phase II. In the work^20^, in particular, the proposed model predicted whether a clinical trial would be successfully completed or not. Manem^21^ developed a new approach for working with clinical trials based on network science, which potentially would better capture the complex nature of the disease. The machine learning models are also widely used in prognosis, prevalence, and mortality analysis of COVID-19^22,23^.

Complex relations between news, drugs, and financial networks allow researchers to investigate the company and market operating principles on a system-wide level rather than case-specific. Wan^24^ employed natural language processing approaches to clarify the impact of news sentiment in the network of companies on the financial market. It turned out that positive news on some companies also positively influenced their neighbors in the co-occurrence network. There^25^ was a new method proposed to assess network similarity, which may be an indicator of causal links. An early warning system was considered a practical application for understanding interconnections between announcements and market response. The design of such systems based on machine learning algorithms was discussed^15,16^.

In this research, our goal is to forecast the market value change caused by the release of clinical trial results, considering the pharmaceutical industry’s peculiarities. To accomplish this, we meet the following objectives as the study progresses.

- Explore one of the biggest clinical trial announcement datasets, including 5436 announcements of 681 public companies.
- Prove statistical evidence on the reasonableness of numerical evaluation of announcement impact.
- Develop a unified framework for efficient preprocessing of clinical announcement data.
- Obtain high-quality prediction for price change range.

## Methods

**Figure 1:**
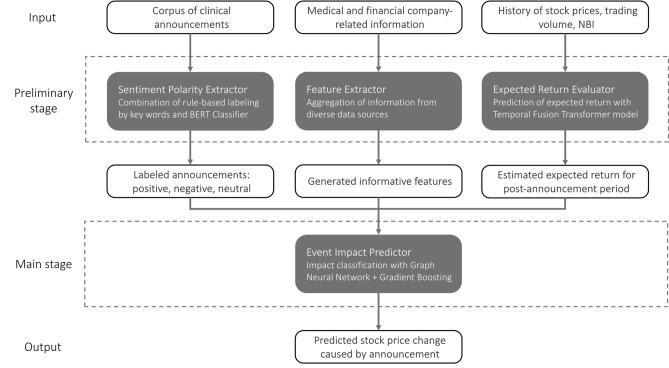
High-level pipeline for the prediction of stock price change induced by the clinical trial results. The preliminary stage consists of the data preparation, including the formation of the feature space and estimation of the expected return needed for the target variable calculation. The main stage includes the core classification model for the prediction of the price change range.

In this section, we discuss the major components of the whole pipeline for change range prediction of company market value. The schematic representation of the pipeline is provided in Fig. 1. There are two stages in the problem-solving process: a preliminary stage and a main one. The preliminary stage serves as a data preprocessing. It focuses on the formation of representative feature space and the evaluation of the expected return, which is required to calculate the target variable. In particular, at the preliminary stage, we do the following. Firstly, we provide details on how we define the critical characteristic of the announcement, namely, its sentiment polarity. Secondly, we describe other generated attributes related to the market, company, and announcement in general. The identified announcement polarity and other extracted features constitute the entire feature space, which we design for the training machine learning model. Thirdly, we explain the approach to calculating the expected return necessary for obtaining the target measure of announcement impact. The main stage consists of the core classification model that makes its prediction of the price change range based on the input feature space prepared at the preliminary stage.

### Statistical tests for announcement impact justification

**Figure 2:**
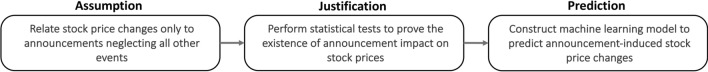
Visualization of logic for correlating the announcement events with the stock price changes. The essential points consist in making an assumption, performing statistical tests, and creating a predictive model.

In this study, we consider events related exclusively to the trial test promulgation, neglecting other events nearby in time. To justify the impact of the trial test announcements on the company’s market value, as well as to highlight the motivation for the construction of the predictive model, we perform statistical tests. The overall logic behind our research is demonstrated in Fig. 2.

In our statistical investigation, we first compare the distribution of announcement-induced price changes with the normal distribution to expose conceptually important asymmetries. For that, we perform the Kolmogorov–Smirnov normality test with a p-value of 0.05. For the next stage, we are naturally interested in comparing price change distributions in the announcement and non-announcement conditions. For this purpose, we perform a rank non-parametrical statistical Mann–Whitney U test with a p-value of 0.05. The non-announcement samples are generated from 20-day period before the announcement. The null hypothesis in this test states that announcement-caused distributions do not differ from non-announcement ones.

### Sentiment polarity extraction from clinical announcements

As we aim to predict the market value change induced by a public clinical announcement, the identification of its sentiment polarity plays a pivotal role. We emphasize three polarity groups: the corpus of positive announcements (e.g., clinical trial approvals), the corpus of negative announcements (e.g., clinical trial termination, negative results), and finally the corpus of neutral announcements. To define the sentiment polarity, we process the collected corpus of historical releases in three phases:

1. We compose the initial dictionaries with keywords, which reflect the announcement polarity. Examples of positive words are “approve”, “meets”, and “show”. As indicators of the negative polarity, we set “halted”, “no differentiation from placebo”, “did not reach”, and “failed”. Then, we construct a rule-based announcement mark-up by checking for the presence of the mentioned words.
2. We take the pre-trained bidirectional encoder representations from transformers (BERT)^26,27^ model and train it on all FDA trial result texts labeled with a rule-based method. After that, the trained BERT model is used to classify the announcements and reveal the examples on which it makes mistakes. Dictionaries are complemented with additional keywords extracted from mistakenly classified announcements.
3. We create final announcement mark-up by leveraging updated dictionaries. The examples of words that are added with the help of the BERT model are “demonstrate”, “potential”, “accepted”, “encouraging” for the positive case and “terminated”, “discontinued”, “insufficient”, “paused” for the negative case.

Thus, we exploit the pre-trained BERT model as a support tool for more accurate mark-up. We deem that labeling announcements by keywords is a reasonable approach because the message of most announcements is delivered in a pronounced way. The obtained sentiment polarity serves as one of the features of the core classification model. We make a choice in favor of a BERT-based model^26^, as this class of architectures shows state-of-the-art performance on various natural language processing benchmarks in sentiment analysis problems^28–31^.

Separately, within the frame of event study, two key announcement representations can be distinguished: sentiment polarity and text embedding. The work^32^ explores the possibility of eliminating the sentiment extraction procedure by direct adoption of relevant text embeddings for stock price prediction, but the idea ends up with no metric gains. Authors claim that despite the seemingly richer contextual information provided by embeddings, the sentiment polarity extraction method demonstrates better performance in event study experiments. Hence, this conclusion motivates us to use a sentiment extractor as a separate instrument.

### Aggregation of relevant information

Following the common machine learning paradigm of data preprocessing, we aim to construct a feature space that encompasses valuable information from various data sources to get a top-quality predictive solution. In our problem statement, the feature space is defined as a set of parameters that impact the stock price change after the announcement takes place. Hence, we generate new features that belong to one of the three domains: market, company, or announcement. Market and company features cover the trade and financial aspects of the company’s operation. The announcement features incorporate sentiment polarity and medical-related attributes. The overall view of the constructed feature space is as follows.

- *Market features* These features describe the stock prices and index dynamics. Namely, they include the mean number of trading volume peaks per year, duration of the last trading volume peak, NASDAQ biotechnology index (NBI), and stock price trend for the last 30 days before the event and the previous 30-day trend (from 60 to 30 days before the event).
- *Company features* We extract the company features from the annual reports. This does not consider quarter reports because not all companies publish them. We consider three types of reports to extract valuable information. The first type is the income statement, normalized by “Total Revenue”. The second type of report is the balance sheet, values of which are normalized by “Cash From Operating Activities”. Also, we take such features as “Total Common Shares Outstanding”, “Full-Time Employees”, and “Number of Common Shareholders” without further normalization. The third type is the cash flow. We normalize it by “Total Equity”.
- *Announcement features* Each pharmaceutical announcement can be associated with a specific nosology. We get the International Classification of Diseases 10th Revision codes (ICD-10 codes) by matching the mentioned diseases in the announcement texts with the particular codes and use them as categorical features. Besides, the extracted sentiment polarity is also referred to as the announcement features.

### Target measure of event impact and evaluation of expected return

The numerical effect of a certain event is usually examined as a difference between the actual return dynamics *R*(*t*) and the expected returns *ER*(*t*) within a post-event period, limited to *T* days after the announcement is made ($t=0$). Thereby, the abnormal return *AR*(*t*) on day *t* after the announcement takes place can be defined as follows $AR(t)=R(t) - ER(t)$. To measure the integral effect within a given post-announcement period (0, *T*], we use normalized cumulative abnormal returns (NCAR). NCAR is defined by the following formula: $NCAR_T = \int _0^T AR(t) dt / \int _0^T ER(t) dt$, in which normalization is required to unify the target feature over announcements for various companies. $NCAR_{20}$ is chosen as a target value we aim to predict for upcoming clinical releases. The motivation for taking the window width of the post-announcement period equal to $T=20$ stems from the values of reacting period. More information on selecting the post-announcement period is given in Supplement.

The expected return dynamics prediction, as a time series forecasting problem, is of high importance, as it directly contributes to the target value. In our research, we examine a Linear Regression (LR) as one of the most spread models in classical expected return evaluation^13,33,34^, Long-Short Term Memory (LSTM) as a frequently exploited model in market time series processing^35,36^, and finally Temporal Fusion Transformer (TFT)^37^ as the-state-of-the art model that shows high-level metrics on several benchmarks^37^, specifically excelling in stock market forecasting^38^. We compare performance metrics for all models to choose the one with the highest quality. Eventually, we show (see “Results” section) TFT outperforms all other models, so we focus on it for the rest of our work.

TFT is a model with an attention-based architecture that promotes capturing complex temporal relationships. In our settings, TFT comprises 1 LSTM layer^39^ and 3 attention heads^40^. The model’s architecture makes it possible to consider various aspects of trading without manually entering cause-and-effect relationships. We use mean absolute percent error (MAPE) as a loss function for our model because of its independence from the absolute stock price. The model is pre-trained on the whole trading history of the company excluding near announcement dates through the use of a window comprising 30 consecutive days for training and 20 days for prediction. The positions of this window in the timeline are selected arbitrarily. After that, for each event, we train the model on 90 days of the trading history before the event date, shifting the same window by one day each time in order to allow the model to understand short market trends. The whole time series is normalized by the first value in it.

The trading history we process with the TFT model consists of stock price, trading volume, and NBI. All of them allow estimating the expected return more precisely, taking into account implicit underlying factors. The use of trading volume facilitates accounting for information on hidden events, such as an announcement of dividends, quart reports, and many others. NBI is used as a global indicator in the pharma market. Within the NBI calculation, more than 100 companies are accounted for. This factor allows not to concentrate on one company but to evaluate the whole pharma market.

### The classification model for price range prediction

To predict the influence of FDA announcements on the stock price, we train the model that matches the input feature space composed of information on market, companies, and announcements with the target impact measure, $NCAR_{20}$. In this work, the concepts of NCAR and price change are used interchangeably. Due to the insufficient number of regarded clinical announcements, a pure regression model for the stock prices does not provide acceptable quality. Therefore, we reduce the problem dimension by transforming a regression setting into a classification. Instead of price change prediction, we predict the range of price change. The proposed problem statement that involves operating with the ranges grants knowledge of the price change’s sign and amplitude. That is why the reformulated problem practically remains a significant concern in terms of risk evaluation.

As a core classifier, we test Gradient Boosting (GB)^41^ and Random Forest (RF)^42^, tree-based methods that stand out from other models in their performance on tabular data^43,44^. Data consisting of the pharma announcements are notable for the presence of intrinsic relationships, which can be represented in the form of a graph. The inclusion of such type of information into the classification process can potentially improve the predictive quality of the model. We use a Graph Convolutional Network (GCN)^45^ that is effective for extracting valuable features from a graph of interconnections. GCN aims to learn the representations that will encode local graph structure and features of nodes. At the start, we solve our classification problem using GCN exclusively. The graph is constructed in accordance with the following principles. There is an edge between two events in the graph if (i) the earlier event happens with the same company or nosology as another one, and (ii) the time period between events is less than 1 year. The architecture of GCN consists of 3 fully connected layers and 2 graph convolution layers^46^. Each node in the graph is represented as a vector that encapsulates the features of the corresponding announcement. The key idea behind the leveraged graph model is that the currently considered event updates its vector representation by exchanging the information between the neighboring nodes. The motivation for taking the time period for establishing the event connections to be less than 1 year is in the optimal number of neighbors of the considered event, which is not too small to produce an uninformative neighborhood and not too big to cause event overlapping and muting. The purpose of GCN is to classify the price change caused by the announcement, so its output is the probability of belonging to a particular class. The classification is done proceeding from the resulting node representations. After that, tree-based models take the composed feature space as input together with the output probabilities of the GCN model to get the final class probabilities. Such a setting is a clear representation of the classification problem on tabular data. Adopting the graph in conjunction with GB or RF promotes the capturing of event interconnections and enhances the overall predictive quality.

## Results

### Sentiment polarity evaluation

Sentiment polarity is the characteristic of an announcement that predominantly determines the subsequent market response. We leverage the power of the pre-trained BERT model in our labeling process to make the final mark-up more reliable. First, we juxtapose the answers on sentiment polarity obtained from the rule-based approach with the initial keywords and the trained BERT model. Second, we analyze the differently labeled announcements and retrieve additional keywords from them to update the rule-based mark-up. The comparison of the number of divergences and coincidences in answers before and after the keyword update is provided in Table 1. As we can see, the number of not matching answers of the rule-based method and the BERT model decreases after the keyword supplement. In addition, we observe a drop in the number of announcements labeled as neutral, since more announcements become emotionally charged as positive and negative. Eventually, the rule-based labeling and BERT predictions become more coherent, which leads to a higher quality of the mark-up.

Originally, we take all the FDA announcements that are available for the considered five-year period. This amounts to 5436 releases with the following class distribution after the discussed procedure of sentiment polarity identification: 1595, 798, and 3043 of positive, negative, and neutral announcements, respectively. However, we exclude all events related to private companies because of the inaccessibility of their stock prices. Thus, for further analysis, we have 1105 positive and 549 negative announcements at our disposal.

**Table 1:** Comparison of the sentiment polarity mark-ups derived from two methods, rule-based and trained BERT, before and after the keyword update.

	# Divergences	# Coinciding positives	# Coinciding negatives	# Coinciding neutrals
With the initial keywords	207	1447	445	3337
With the updated keywords	66	1562	765	304

The number of divergences represents the number of announcements labeled differently by these methods. Whereas, for example, the number of coinciding positives reflects the number of announcements that are positive from the perspective of both methods.

### Outcomes of announcement impact analysis with statistical tests

**Figure 3:**
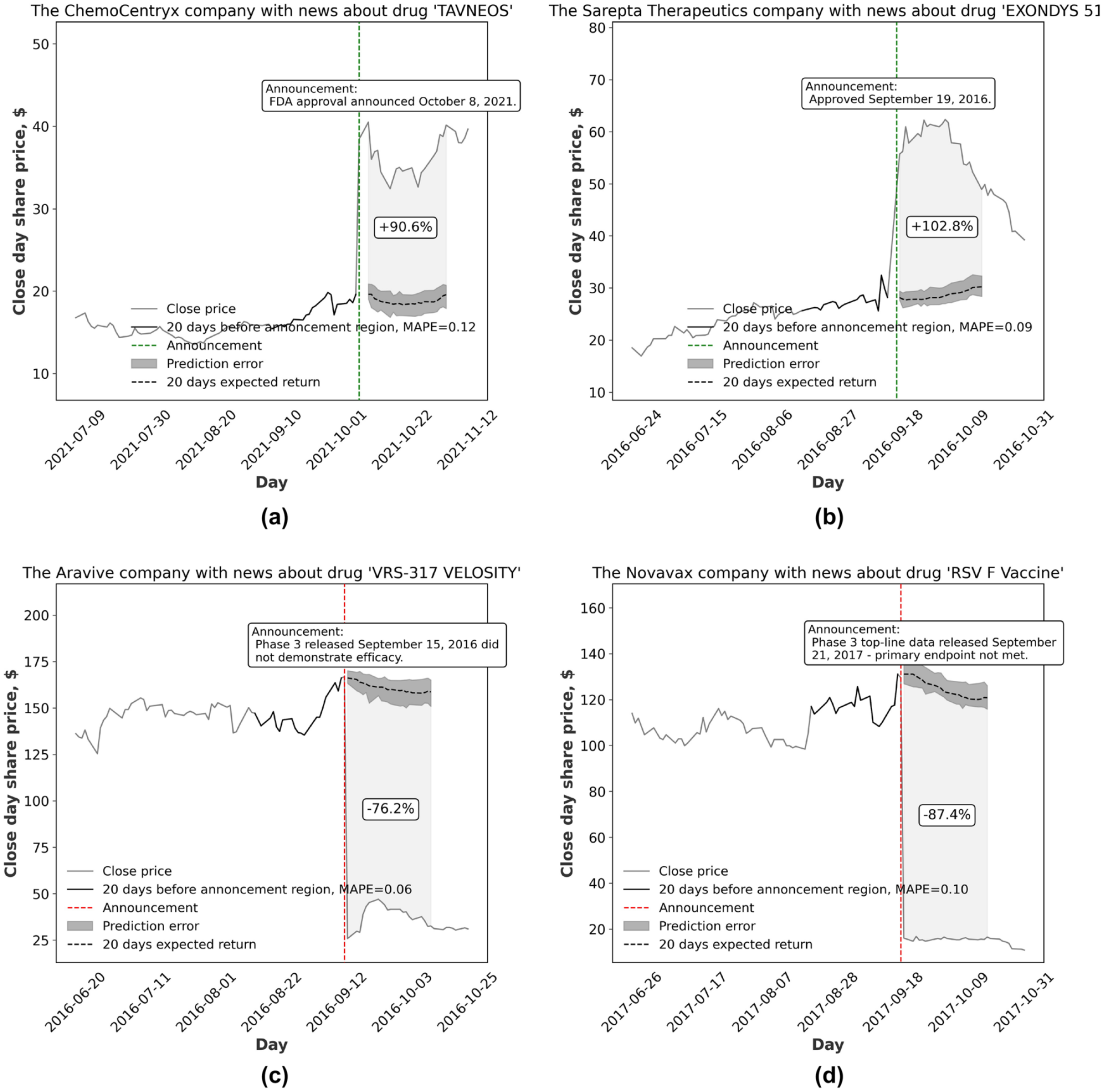
Representative examples of the stock price changes according to the FDA trial clinical results (both positive (**a,b**) and negative (**c,d**) announcements are examined). (**a**) Chemocentryx Announces FDA Approval of Tavneos™ (Avacopan) in Anca-Associated Vasculitis. (**b**) Sarepta therapeutics announces fda accelerated approval of exondys 51™ (eteplirsen) injection, an exon skipping therapy to treat duchenne muscular dystrophy (dmd) patients amenable to skipping exon 51. (**c**) The Resolve™ trial, a Phase 3 trial of our RSV F Vaccine in 11,856 older adults (60 years of age and older), did not meet the pre-specified primary or the secondary efficacy objectives, and did not demonstrate vaccine efficacy (Novavax announces topline rsv f vaccine data from two clinical trials in older adults). (**d**) Announced that the VELOCITY Phase 3 clinical trial of somavaratan in pediatric growth hormone deficiency (GHD) did not meet its primary endpoint of non-inferiority (Versartis announces phase 3 velocity trial of somavaratan in pediatric growth hormone deficiency did not meet primary endpoint).

In some cases, the clinical results announcements can have an extreme effect on the company’s financial position. The examples of the most influential clinical events, accompanied by the significant stock price change, are demonstrated in Fig. 3.

**Figure 4:**
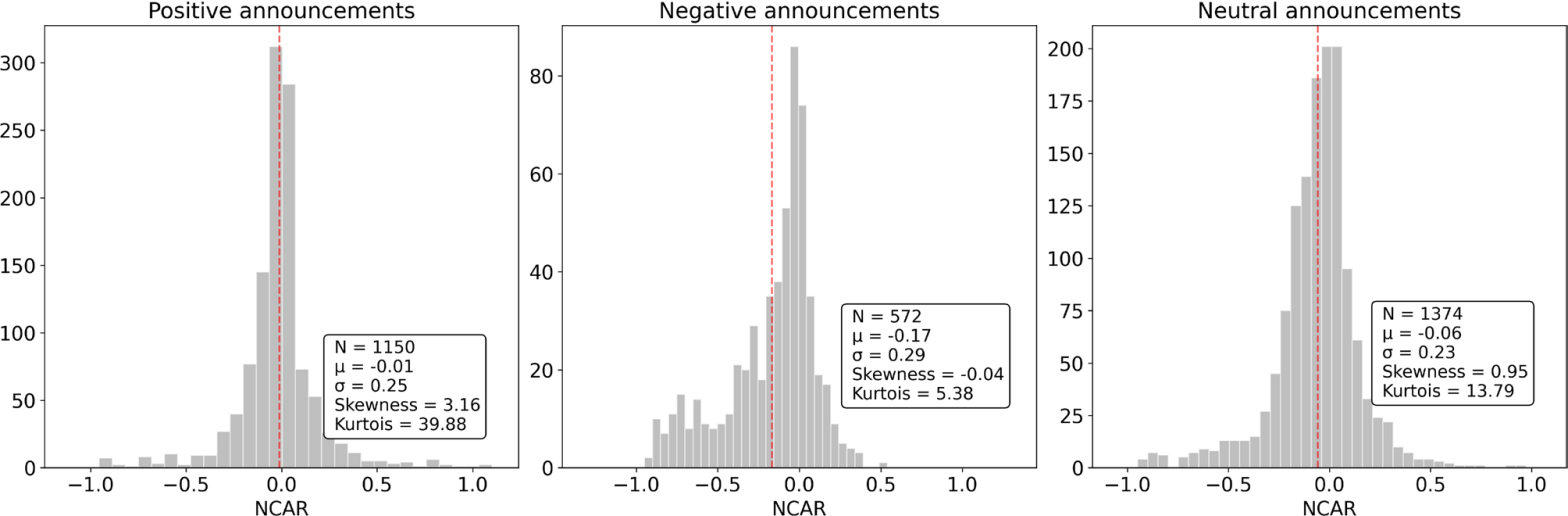
The price change distributions ($NCAR_{20}$) after the FDA announcement with positive, negative, and neutral contents takes place. The statistical parameters are provided in the boxes (number of events *N*, mean value $\mu$, standard deviation $\sigma$, skewness, and kurtois coefficients). The mean values are depicted with red dashed lines.

Our metric for the cumulative characteristic of the price change is $NCAR_{20}$. The obtained $NCAR_{20}$ distributions for the announcements of different sentiment polarities are shown in Fig. 4. As a result of the Kolmogorov–Smirnov normality test for each announcement group, we get a p-value equal to $7 \times 10^{-9}$ for the negative announcements, a p-value equal to $10^{-34}$ for the positive announcements, and a p-value equal to $3 \times 10^{-16}$ for the neutral announcements, which indicates an abnormality of distributions. Indeed, it can be seen from Fig. 4 that there are the abnormality and asymmetry in distributions related to the considered polarity groups. Meanwhile, we observe a significant number of negative announcements with positive $NCAR_{20}$ values, and vice versa. This mismatch indicates that, for instance, positive news (e.g., Phase III acceptance) does not necessarily imply a positive impact on the stock prices. The negative announcements have a rather less mismatch ratio (most of the negative announcements are indeed connected with the negative stock price changes). The inconsistency between the announcement sentiment polarity and actual price change can be explained by the presence of other contributing factors (e.g., financial reports, merging or acquisition announcements, etc.) that are not considered in the frame of this research. A deeper inconsistency analysis is provided in Supplement.

Upon the disclosure of the non-normality of events distributions, we continue with Mann–Whitney U test for the awareness of possible distinction from the non-announcement state. The p-value in the U test for comparing positive and non-announcement distributions is equal to 0.34. This means that the null hypothesis can not be rejected. In the U test with negative and non-announcement distributions, the obtained p-value is equal to $2 \times 10^{-13}$. In this case, the null hypothesis can be rejected.

### Impact analysis of company background on stock prices

**Figure 5:**
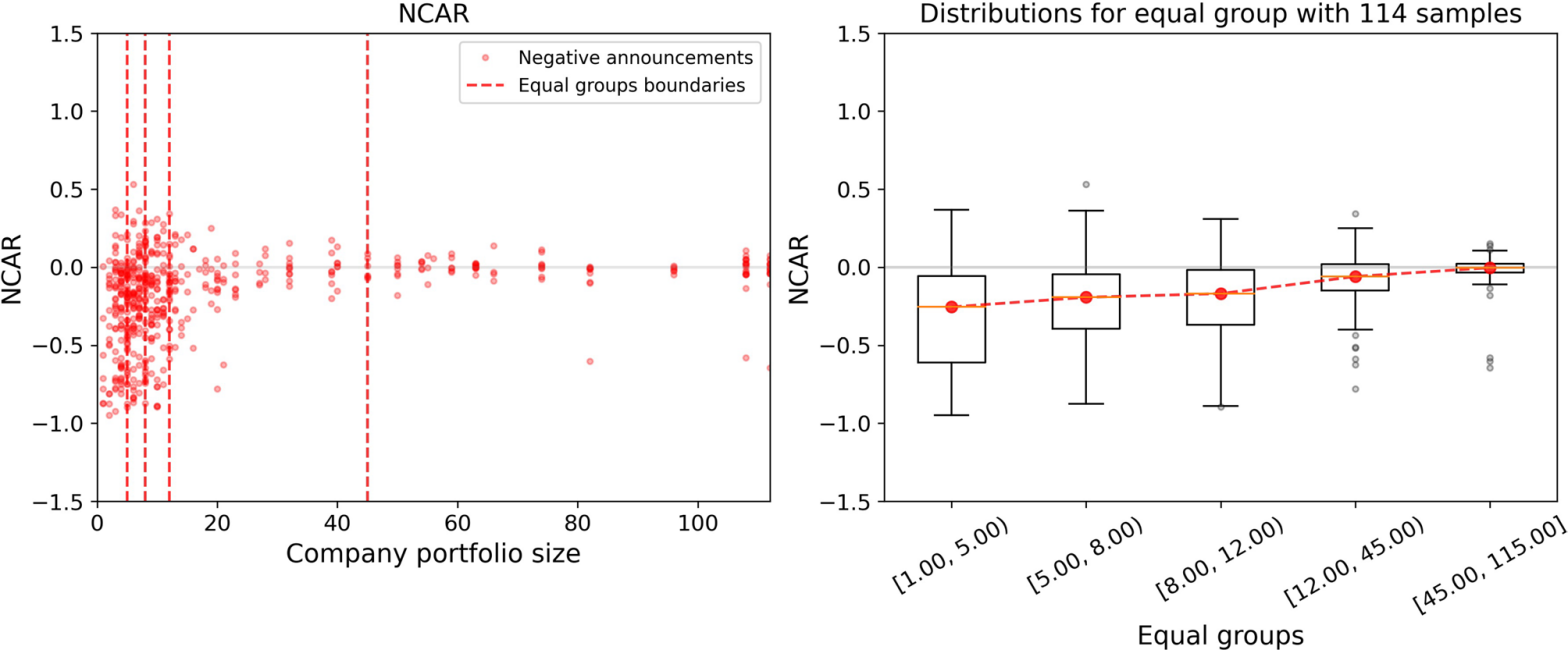
Dependence of the price changes over equal groups on size of the company drug portfolio for the negative announcements. In the left part, the red dashed lines divide announcements into equal groups (with the same number of announcements inside). In the right part, the red dashed line goes through the median values of each group’s stock price changes.

**Figure 6:**
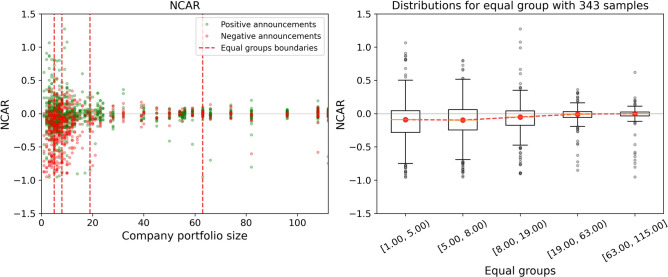
Dependence of price change over equal groups on the company’s drugs portfolio size for negative and positive announcements. In the left part, the red dashed lines divide announcements into equal groups (with the same number of announcements inside). In the right part, the red dashed line goes through the median values of each group’s stock price changes.

One of the key characteristics of the pharmaceutical company’s background is its drug portfolio size. This feature indicates the number of products that are supplied to the market. The portfolio size represents company scope and capacity, depending on which the reaction to the announcements of the same polarity may differ. In Figs. 5 and 6, the price change is given for the various number of products in the portfolios of the examined companies. This demonstrates that small companies are more susceptible to any announcements in terms of their stock prices, whereas the positions of big companies are more robust. Moreover, for small companies, it is more probable to get a negative stock price change as a reaction to some event. More information on the dependence of stock price change on the company characteristics is given in Supplement. To sum up, we observe that the patterns of price changes can depend significantly on the company’s background, which is the strong motivation for the generation of diverse features for the predictive model.

### Expected return evaluation results

One of the essential steps is an evaluation of the expected return, which is necessary for the calculation of an event’s impact on stock prices. The time series is handled using the TFT model. To get a sense of its performance within our problem setting, we estimate the mean value of errors on 20-day periods before events. To do so, we train TFT on data from 110 to 20 days before events using the same sliding window described in the “Methods” section. After the model training phase, we predict stock prices for 20-day periods before events. We obtain MAPE of 0.07 for the dataset with positive and negative events. We also measure the metrics for predictions made by LR and LSTM. They significantly underperform TFT, showing MAPE equal to 0.14 and 0.11, respectively. To conclude, the TFT model shows good predictive quality and can be used for the appropriate evaluation of the expected return in a post-event period.

### Results on classification task for price change

**Figure 7:**
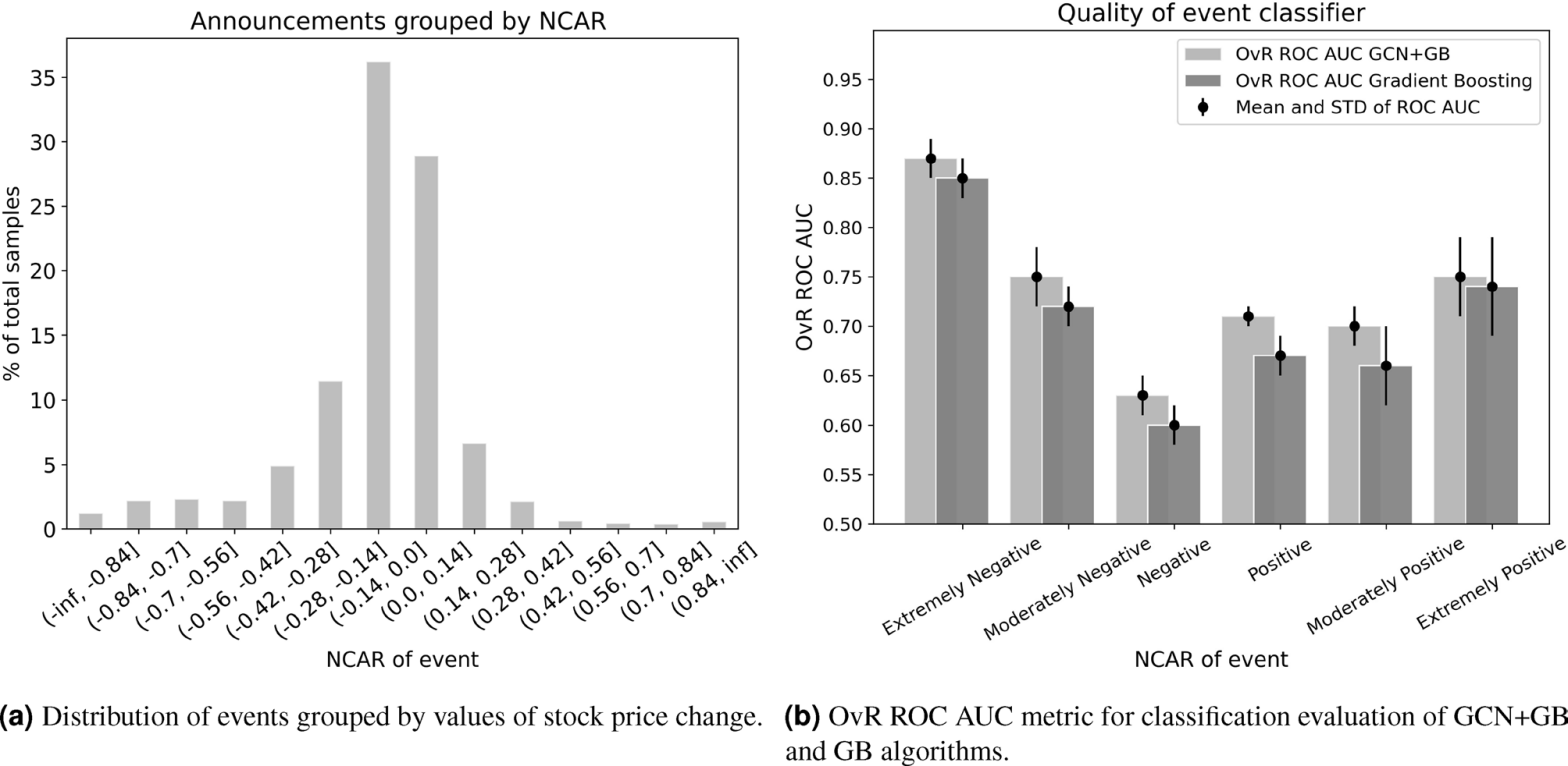
Samples distribution and classification metrics according to price change ranges.

Market conditions, company characteristics, announcement polarities and content, along with event relationships collected in one place make it possible to predict the price changes caused by the announcements. By setting our problem statement as a classification task, we categorize the stock price change values into six classes: Extremely Negative, Moderately Negative, Negative, Positive, Moderately Positive, and Extremely Positive. The number of classes is conditioned for three reasons. Firstly, it is important to know a sign of reaction. Secondly, we need to get a clear understanding of the impact amplitude. Thirdly, each class needs to be representative. Taking into consideration the indicated issues and the fact that our MAPE for the expected return evaluation is about 7%, we define the range of price changes that belong to one class equal to 0.14. Figure 7a shows that a small number of events cause price change greater than 0.28 in amplitude. Thus, due to the third reason, we combine announcement responses with price changes of more than 0.28 into one class.

For the experiments, we split our dataset with the announcements in a stratified way by 10 times with the train and test subsets of 67% and 33%, respectively. As the classification model, we use the combination of GCN and GB. This allows us to improve the resulting classification quality by the combination of different types of data representation and the incorporation of the event interconnections. To figure out the advantage of adopting GCN, we calculate metrics for the case when prediction is made only with GB. One-vs-Rest (OvR) ROC AUC is taken as the main metric for the evaluation of model performance. The results are presented in Fig. 7b and Table 2.

**Table 2:** Events characteristics depending on the caused stock price change and model performance metrics.

Class name*	Extremely	Moderately			Moderately	Extremely
	Negative	Negative	Negative	Positive	Positive	Positive
Stock price change range	($- \,\infty$, − 0.28]	(− 0.28, − 0.14]	(− 0.14, 0]	(0, 0.14]	(0.14, 0.28]	(0.28, + $\infty$)
Number of events	211	189	599	478	110	67
Positive events**	72	106	421	366	83	57
Negative events**	139	83	178	112	27	10
OvR ROC AUC for GCN+GB	$0.87 \pm 0.02$	$0.77 \pm 0.03$	$0.63 \pm 0.02$	$0.71 \pm 0.01$	$0.70 \pm 0.02$	$0.75 \pm 0.04$
OvR ROC AUC for GB	$0.85 \pm 0.02$	$0.72 \pm 0.02$	$0.60 \pm 0.02$	$0.67 \pm 0.02$	$0.66 \pm 0.04$	$0.74 \pm 0.05$
Welch’s t-test p-value***	0.09	0.05	0.002	$5.4 \times 10^{-5}$	0.02	0.65

*According to the value of price change.

**According to the sentiment polarity of the announcement.

***Welch’s t-test p-value for equality of GB and GCN+GB metrics distributions.

As it can be seen from Table 2, the best model GCN+GB achieves OvR ROC AUC greater than 0.7 for all classes, excluding the Negative class. The model distinguishes the Extremely Negative class most accurately, demonstrating ROC AUC score of 0.87. The total weighted OvR ROC AUC for GCN+GB is equal to 0.71. In Table 2, we also present the results of Welch’s t-test with a p-value of 0.05 for the comparison of metric means for GB and GCN+GB models. Welch’s t-test is an adaptation of Student’s t-test but is more reliable when the two distributions have unequal variances. The null hypothesis states that the two population means are equal. We can not reject this hypothesis for Extremely Negative, Moderately Negative, and Extremely Positive classes when comparing the performances of the two models. Figure 7b clearly demonstrates the efficiency of using GCN, which integrates interaction between events due to construction specificity. There is an especially tangible impact on the results for the Positive class. We provide a fuller comparison of different machine learning models, namely GCN, GB, RF, and combinations of GCN with GB and RF, in Supplement.

### Analysis of feature importance

**Figure 8:**
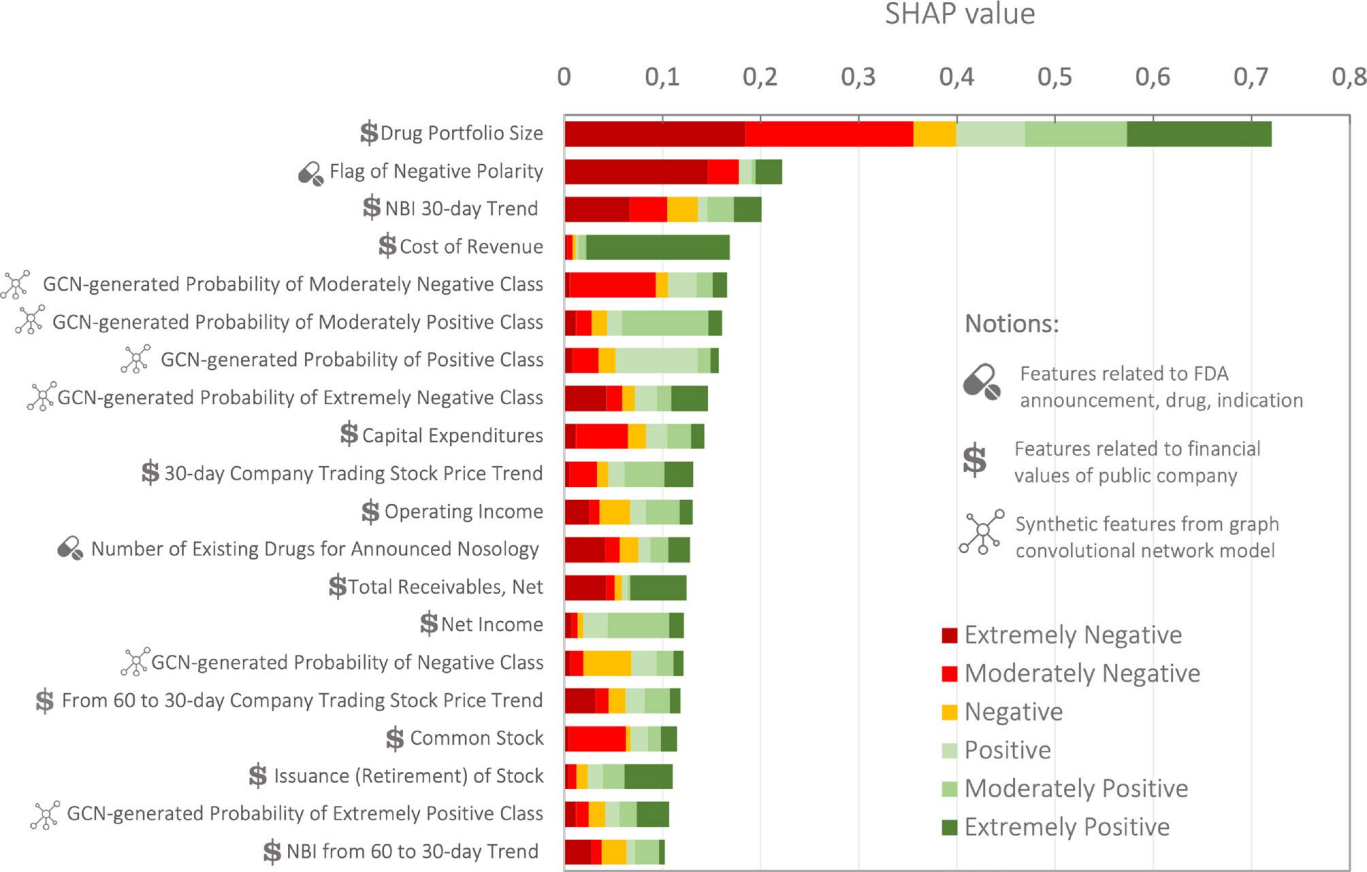
Top-20 of the most important features for Gradient Boosting Classifier according to SHAP.

We manage to train the core classification model (GCN+GB) in a way that provides satisfactory performance metrics. For this reason, we can get a credible assessment of the importance of features utilized for prediction construction. We leverage the SHAP^47^ method for this purpose. Feature importance is estimated when using GB, which takes constructed feature space and the class probabilities from GCN as input. The resulting distribution is provided in Fig. 8.

The “Drug Portfolio Size”, “Flag of Negative Polarity”, and “NBI 30-day Trend“ features occupy the first three positions in terms of importance. Notably, all the features generated by GCN are located in Top-20. Many top features are associated with the indicators from reports or statistics on the trend data. “Number of Existing Drugs for Announced Nosology” stands out over other features, as it is designed using hybrid information from medicine and the announcement.

## Discussion

In this work, we provide a framework for operation with the pharma stock market and its reaction to the trial result announcements, handling one of the biggest datasets of 5436 releases by 681 pharmaceutical companies. Importantly, we statistically prove that there is an influence of the announcements on the stock price, thereby confirming the validity of our problem statement. The analyzed distributions disclose that the responses to the positive and negative announcements are inherently different. In particular, from Fig. 4, we can see that the negative events lead to a greater and more definitive impact on the share prices than the positive ones. Specifically, the negative events cause more significant asymmetry in the price responses, expressed in sharp financial losses in some cases. This kind of effect is reported in many studies and is usually explained by overconfidence in future success^48^. Such overconfidence implies the presence of positive expectations in the recent share prices. Hence, the prices should not change much if the results are indeed positive. Also, a stronger reaction to negative announcements can be substantiated by the ambiguity of the future state of affairs after positive events in comparison with the certainty after negative ones^49^. Negative trial results often signify that the treatment under development will not be forthcoming, consequently inflicting a financial hit. Anyway, the sign of the price change effect can not be straightforwardly determined from the announcement polarity.

A further detailed examination of available data leads us to the unveiling of the fact that the drug portfolio size of the company is a key characteristic governing the price reaction. In short, the more diversified the company is, the less harmful effect from the negative trial test announcements may be expected. Significantly, we manage to identify the precise risk boundaries. As it can be seen from Fig. 5, the companies with a drug portfolio size varying from 1 to about 5 products experience a strong sensitivity to negative announcements. There is a clearly observed increasing trend of price change medians towards zero with the growth of company diversification. Remarkably, companies with portfolio size greater than 45 have immunity against negative clinical news. The extracted feature importances, as part of the model interpretation technique, support the primacy of the attribute responsible for the company drug portfolio size. This finding is in line with the dissimilar statistics of abnormal returns after the success or failure of clinical trials for companies of different maturity. Abnormal returns are much greater in the case of early biotechnology companies compared to large pharmaceutical companies^50^.

We find out that the incorporation of the GCN-generated attributes noticeably enhances the classification metrics. In the meantime, the prevailing number of GCN by-products occupy top positions in feature importance distribution. All these point to the presence of companies’ interdependencies that should be taken into account to get predictions of higher quality. Such a phenomenon can be characterized as an announcement network effect. The observed network effect aligns with a real-world situation under which the behavior of the particular company is altered by the activities of other pharma players in the market^51^. Another argument for integrating the market context is the high-importance position of such specific feature as the number of existing drugs for the announced nosology.

While conducting the analysis, we notice several seemingly inconsistent outcomes. Firstly, there are no features in the Top-20 that are associated with the clinical trial phase when the announcement is released. However, several studies note that the reaction to product-related events is contingent on its level of development^50,52^. Here it is worth mentioning that feature importance obtained with a specific method should not be blindly followed as it does not provide a conclusive picture. One should understand the limitations of leveraged approach for getting feature importance and treat it as a guide, not the ultimate truth. So, in our case, GCN features and financial characteristics turn out to be more important than the trial phases. Secondly, in reviewing the resulting values of the core classifier metrics, we see that the model shows bad quality for the Negative class ($(- 0.14, 0]$ interval of $NCAR_{20}$) in comparison with other classes, although it contains the highest number of samples. We suppose that the reason lies in a weekly pronounced price change effect which implies more ambiguities. Under such case, inaccuracies in the expected return prediction might cause particularly adverse interference.

Nevertheless, the developed framework for predicting the stock price response implies several assumptions and limitations. The basic assumption is that the stock prices incorporate all relevant information available to market traders. In this case, the stock price is instantaneously affected by newly revealed information related to the examined company^53^. Next, we assume there is no information leakage in a market before the release takes place. It means we set an official announcement as a starting point for the market reaction. It is worth noting that the official FDA announcements could be published at a time when the preliminary results are publicly available. In fact, the stock price of publicly traded pharmaceutical companies tends to react to the clinical trial results before official releases^54^. Furthermore, we isolate the effect of clinical result announcements from the other events. This assumption is the most critical because there are definitely other factors impacting the stock prices (e.g., declaration of dividends, financial report, new product announcement, merging with another company, new CEO announcement, etc.). However, we believe that clinical trials have one of the most pronounced impacts on the producer’s financial state. At the same time, we attempt to account for other non-visible events by tracking the trading volume. Usually, the most active moments of trading correspond to the major events that are not necessarily associated with the company. Finally, we do not examine the prediction of the clinical result itself as it is a self-contained complex task demanding a detailed investigation.

The motivation behind the diversification of employed models BERT, TFT, and GB+GCN is the following. Each unit of the proposed framework aims to solve the particular sub-problem. It is either sentiment analysis, time series prediction, or classification on tabular data with a preliminary processing of graph-structured information. All these subtasks have a distinctive nature and underlying data types. Therefore, for the sake of more optimal performance, we make a choice in favor of differing models in each stage rather than using the same architecture with input–output adaptation. In every case, we opt for the model that proves itself in terms of consistently high prediction quality and robustness within the specific problem type. Additionally, we wish to elaborate on the factors for implementing several fragmented models instead of advancing the general multi-modal one. On the whole, we prioritize a clear and straightforward progression of development phases that responds to the sequential logic of our work plan. Construction of an intricate model will diminish the adaptability, reduce the capabilities for intermediate control, and potentially require more data to be trained effectively.

The principal distinguishing and novel trait of our study is the construction of the framework for making predictions at the intersection of clinical and financial domains with the ability to reveal precious subject-specific insights. To the best of our knowledge, we are the first who consider the problem related to the announcement-induced price changes in the predictive paradigm, whereas the previous works focus on ex-post assessment^13,50^. We believe that the scientific value of our research consists in the feasibility of reusing the proposed approach in other domains due to the easily generalized logic of each framework’s subpart. The main requirement for the reapplication is the presence of two entities: events and associated time series that is substantially influenced by these events. Moreover, each subproblem is solved with the well-proven and established models. From this perspective, the developed framework is robust in terms of transferability on new data but with the discussed structure. Summing up, our findings can be helpful for market researchers, especially those who deal with the pharma market and want to make more rational strategic investment decisions and hedge financial risks. In addition, this work contributes to the event studies and demonstrates the direct application of machine learning tools in the industry.

## Conclusions

In this study, we quantitatively relate clinical trial announcements with the market value change of pharmaceutical companies. In particular, we construct a statistically justified predictive framework for price change evaluation and prove a principal presence of forecast potential for future FDA announcement impacts by achieving the total weighted ROC AUC score greater than 0.7 for historical data. Notably, we are able to predict Extremely Negative, Moderately Negative, and Extremely Positive classes of price change most precisely with 0.87, 0.77, and 0.75 ROC AUC scores, respectively. Since we analyze one of the biggest announcement datasets, we extract reliable relationships between the company background and the price change peculiarities. Our research can constitute a solid basis for the further more sophisticated analysis of the pharma market.

### Supplementary Information

Supplementary Information.

## Data Availability

Market data used in this study are publicly available from Yahoo! Finance. It consists of the opening, closing, highest, lowest prices, and trading volume for every trading day. We take the closing price as the stock price. We consider the whole available trading history that is usually available from a company IPO. Financial reports are also available from Yahoo! Finance for the considered years 2018–2022.The information about the clinical trial results was retrieved from the website www.biopharmcatalyst.com, a research-based portal that provides key catalyst updates for publicly traded biotech and pharmaceutical companies. Extracted data contains short texts of clinical trial announcements, information on the clinical phase, tickers of the companies associated with those trials, and announcement dates.
