# Supplementary material for: New drugs and stock market: a machine learning framework for predicting pharma market reaction to clinical trial announcements
Source: Sci Rep. 2023 Aug 7;13:12817. doi: 10.1038/s41598-023-39301-4 (PMC10406841; doi:10.1038/s41598-023-39301-4)
Supplement: Supplementary file 1 — Supplementary Information. [file 41598_2023_39301_MOESM1_ESM.pdf]

New Drugs and Stock Market: A Machine Learning Framework for Predicting Pharma Market Reaction to Clinical Trial Announcements

Semen BUDENNYY[1,2,*], **Alexey KAZAKOV**[1], **Elizaveta KOVTUN**[1], **and Leonid ZHUKOV**[3]

[1]Sber AI Lab, Moscow
[2]Artificial Intelligence Research Institute (AIRI), Moscow
[3]Higher School of Economics University, Moscow
[*]Corresponding author: sanbudenny@sberbank.ru

Supplementary materials

Post-announcement period calculation

To estimate the duration of the announcement effect, we analyze trading volume peaks in the companies' history. These peaks do not exclusively mean the reaction to the trial results but are rather associated with all events related to the company. We calculate the duration in days of all available trading volume peaks. The results are presented in Figure 1. As we can see, the duration of 90% peaks constitutes 20 trade days. Thus, we take 20 days as the post-announcement time period we are interested in.

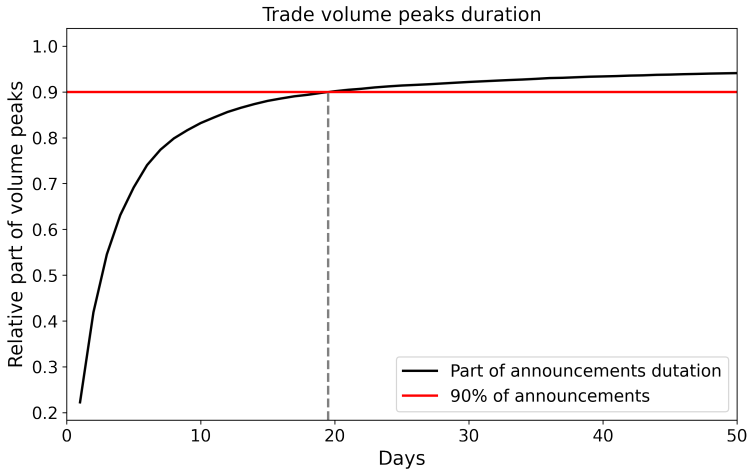

Figure 1. The correspondence between the share of considered trading volume peaks with the peak duration. Redline is the 90% of the total number of peaks.

Other relationships between announcement impact and company characteristics

The dependence of the number of FDA news announcements on the year for the public companies is given in Figure 2. It demonstrates the increase in the number of FDA announcements in open sources by approximately ten times from 2017 to 2021.

Figures 3, 4 and 5 show the dependence of stock price changes caused by announcements on the company age since entering the IPO at the announcement moment, historical volatility, and the company's 30-day stock price trend. Volatility is defined as the relation of the standard deviation of stock price to the median stock price in a 200-day period before the announcement. We can conclude that the younger company is, the more sensitive it is to the announcements. Greater intrinsic company volatility results in more extreme price changes. In addition, if the company trend of a stock price is about zero in the pre-event period, then the induced price change is not significant.

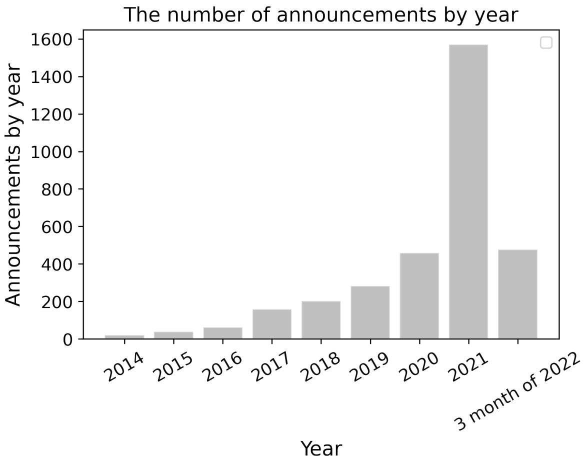

Figure 2. The number of FDA announcements of public companies in different years.

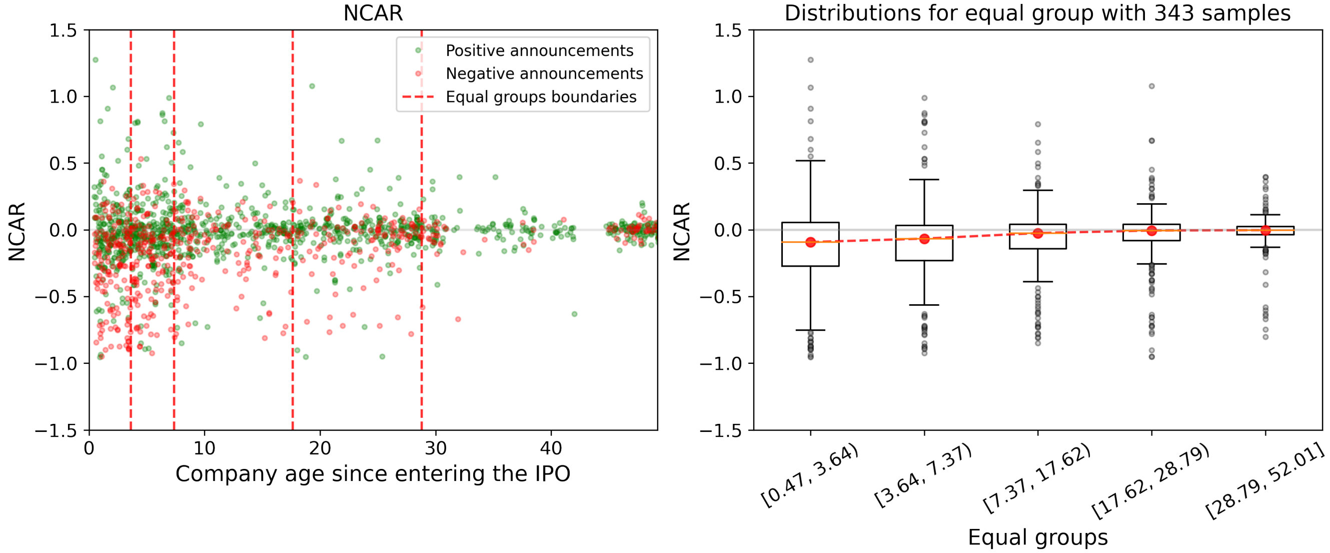

Figure 3. Dependence of the stock price changes on the company age. In the left part, the red dashed lines divide announcements into equal groups (with the same number of announcements inside). In the right part, the red dashed line goes through the median values of each group's stock price changes.

Mismatch analysis between announcement polarities and price change

To realize the mismatch between events sentiment in accordance with FDA announcements and events sentiment in accordance with the actual price change, we build a confusion matrix shown in Figure 6. The sentiment polarity derived from stock price change, namely $NCAR_{20}$, is defined as follows: the event is set to be positive or negative one in accordance with $NCAR_{20}$ if its price change is more than $\sigma_{neut}/2 = 11.5\%$ (σ_{neut} - standard deviation of $NCAR_{20}$ for neutral announcements) or less than $-\sigma_{neut}/2$, respectively. If the price change lies within $[-\sigma_{neut}/2; +\sigma_{neut}/2]$, then we define such event as a neutral one in accordance with $NCAR_{20}$. As a result, the highest rates in the confusion matrix constitute 23.7% and 24.1% and relate to the cases in which negative announcements have negative or neutral price changes.

Comparison analysis of models for classification of price change

Solving the problem of price change classification, we test Gradient Boosting (GB) and Random Forest (RF), which are common machine learning models for tabular data. In addition, we experiment with Graph Convolution Network (GCN) in a node classification setting. We explore model combinations, GCN+GB and GCN+RF, with the aim of enhancing final predictive quality. The results are presented in Table1. The best metrics are achieved with GCN+GB.

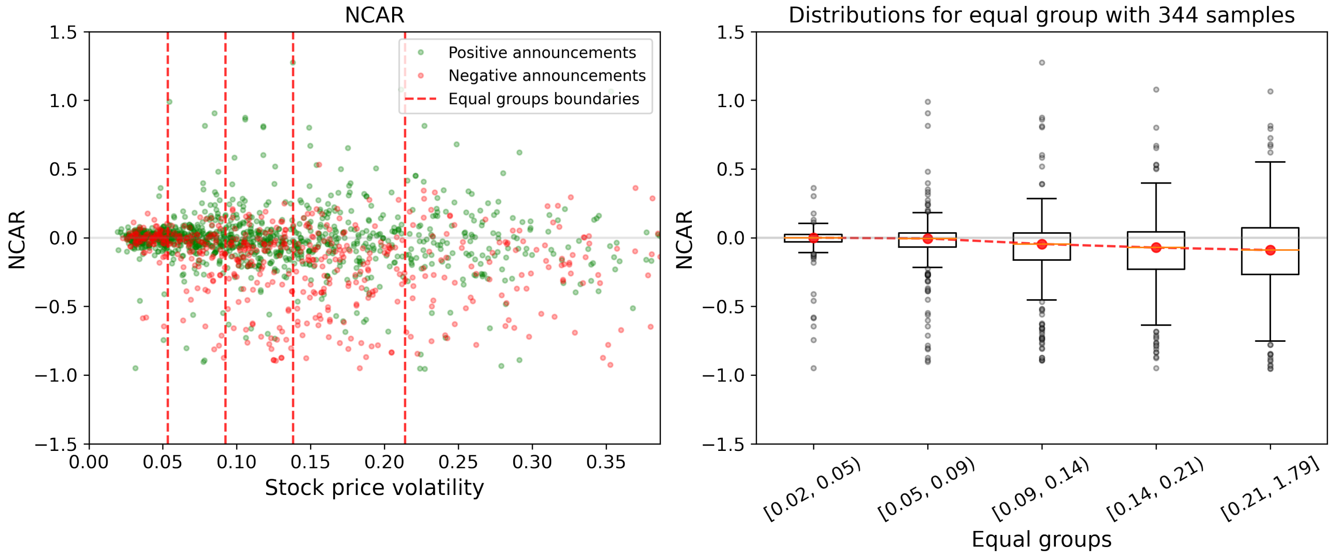

Figure 4. Dependence of the stock price changes on the company volatility. In the left part, the red dashed lines divide announcements into equal groups (with the same number of announcements inside). In the right part, the red dashed line goes through the median values of each group's stock price changes.

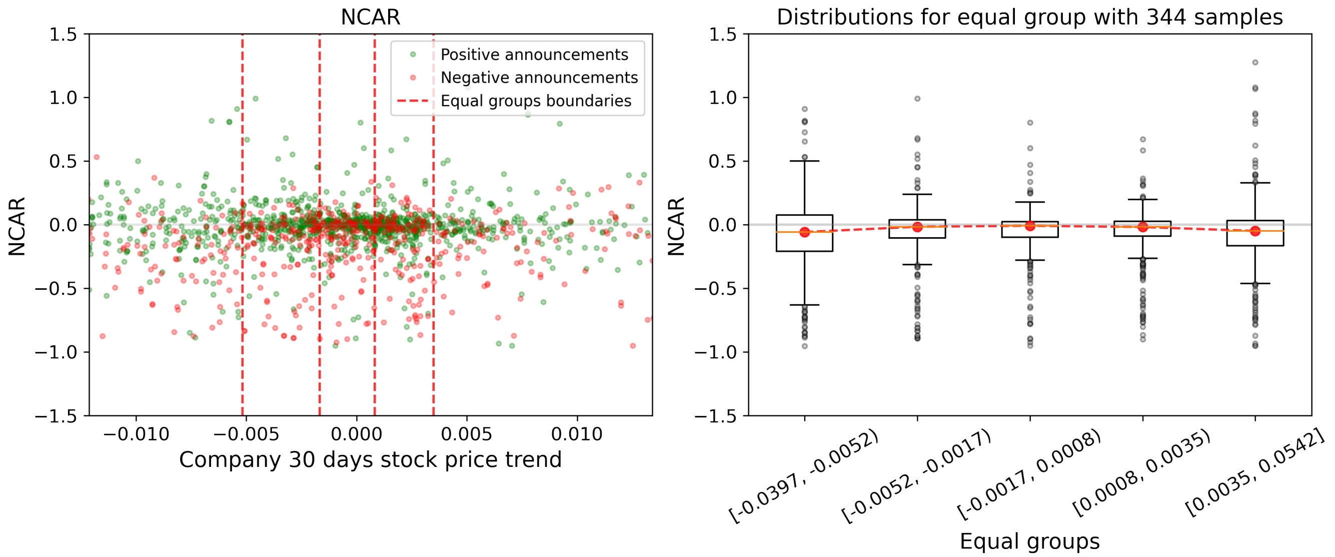

Figure 5. Dependence of the stock price changes on the 30-day trend before the announcement. In the left part, the red dashed lines divide announcements into equal groups (with the same number of announcements inside). In the right part, the red dashed line goes through the median values of each group's stock price changes.

Statement on computational resources and environmental impact

We used a NVIDIA GeForce RTX 2070 SUPER GPU and NVIDIA A100 80GB PCIe GPU to train the classifiers and the BERT model, correspondingly.

This work contributed 1.34 kg and 6 g of equivalent CO_2 emissions during the classifiers and BERT training, respectively. The carbon emissions information was generated using the open-source library eco2AI (https://github.com/sb-ai-lab/Eco2AI).

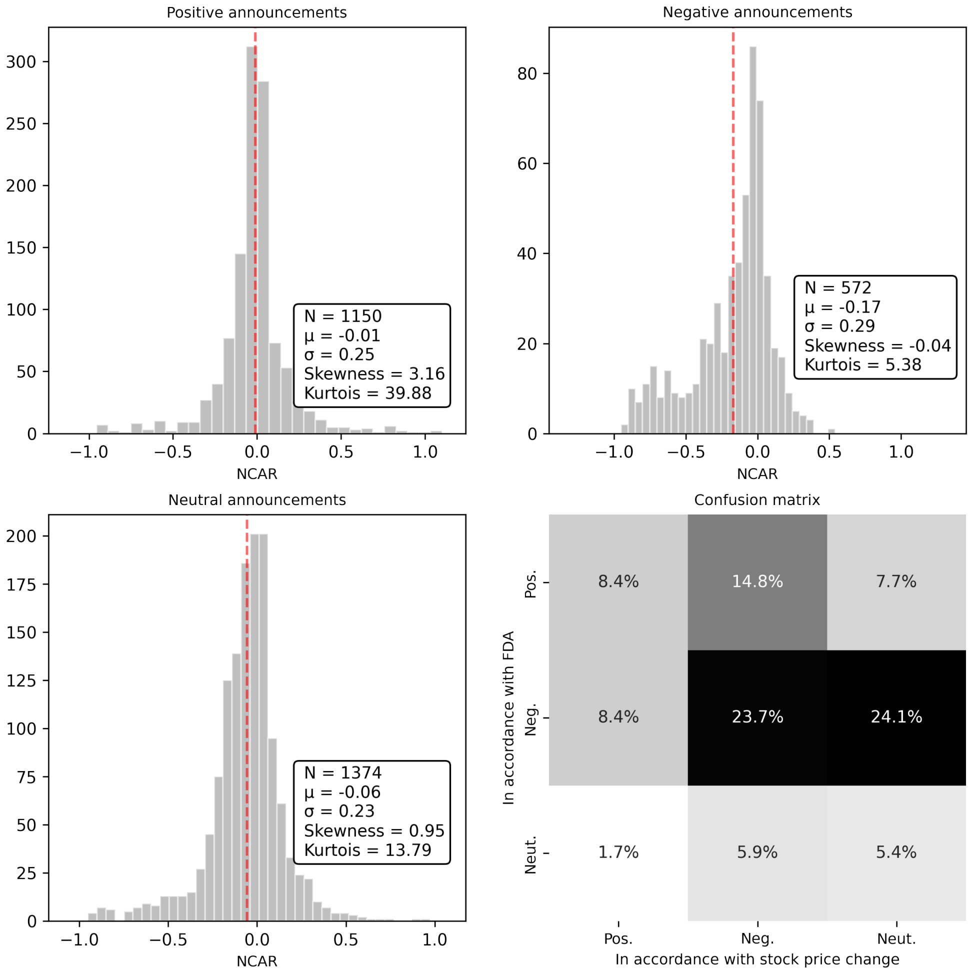

Figure 6. The price change distribution ($NCAR_{20}$) for positive (upper left), negative (upper right), neutral (lower left) FDA news with statistical parameters provided in the boxes (number of events N, mean value μ, standard deviation σ, skewness and kurtois coefficients). The mean value is depicted with the red vertical dashed line. The confusion matrix (lower right) of mismatch between sentiments in accordance with FDA announcements and polarities in accordance with the stock price change ($NCAR_{20}$).

Table 1. Comparison of model metrics in the task of price change classification.

Class name* Stock price change range	Extremely Negative (-∞, -0.28]	Moderately Negative (-0.28, -0.14]	Negative (-0.14, 0]	Positive (0, 0.14]	Moderately Positive (0.14, 0.28]	Extremely Positive (0.28,+ ∞)
GCN	0.60 ± 0.04	0.64 ± 0.04	0.53 ± 0.02	0.61 ± 0.02	0.54 ± 0.04	0.50 ± 0.06
GB	0.85 ± 0.02	0.72 ± 0.02	0.60 ± 0.02	0.67 ± 0.02	0.66 ± 0.04	0.74 ± 0.05
RF	0.82 ± 0.02	0.70 ± 0.02	0.62 ± 0.01	$\mathbf{0.71 \pm 0.01}$	0.68 ± 0.05	$\mathbf{0.75 \pm 0.02}$
GCN+GB	$\mathbf{0.87 \pm 0.02}$	$\mathbf{0.77 \pm 0.03}$	$\mathbf{0.63 \pm 0.02}$	$\mathbf{0.71 \pm 0.01}$	$\mathbf{0.70 \pm 0.02}$	$\mathbf{0.75 \pm 0.04}$
GCN+RF	0.83 ± 0.02	0.72 ± 0.02	0.62 ± 0.01	0.70 ± 0.01	0.67 ± 0.04	$\mathbf{0.75 \pm 0.03}$

* According to the value of price change
